# Modeling optimal reopening strategies for COVID-19 and its variants by keeping infections low and fixing testing capacity

**DOI:** 10.1371/journal.pone.0274407

**Published:** 2022-11-09

**Authors:** Mackenzie Dalton, Paul Dougall, Frederick Laud Amoah Darko, William Annan, Emmanuel Asante-Asamani, Susan Bailey, James Greene, Diana White

**Affiliations:** 1 Mathematics Department, Clarkson University, Potsdam, NY, United States of America; 2 Biology Department, Clarkson University, Potsdam, NY, United States of America; Texas A&M University College Station, UNITED STATES

## Abstract

Since early March 2020, government agencies have utilized a wide variety of non-pharmaceutical interventions to mitigate the spread of COVID-19 and have struggled to determine when it is appropriate to return to in-person activities after an outbreak is detected. At many universities, fundamental issues related to understanding the spread of the disease (e.g. the transmission rate), the ability of administrators to respond quickly enough by closing when there is a sudden rise in cases, and how to make a decision on when to reopen remains a concern. Surveillance testing strategies have been implemented in some places, and those test outcomes have dictated whether to reopen, to simultaneously monitor community spread, and/or to isolate discovered cases. However, the question remains as to when it is safe to reopen and how much testing is required to remain safely open while keeping infection numbers low. Here, we propose an extension of the classic SIR model to investigate reopening strategies for a fixed testing strategy, based on feedback from testing results. Specifically, we close when a predefined proportion of the population becomes infected, and later reopen when that infected proportion decreases below a predefined threshold. A valuable outcome of our approach is that our reopening strategies are robust to variation in almost all model parameters, including transmission rates, which can be extremely difficult to determine as they typically differ between variants, location, vaccination status, etc. Thus, these strategies can be, in theory, translated over to new variants in different regions of the world. Examples of robust feedback strategies for high disease transmission and a fixed testing capacity include (1) a single long lock down followed by a single long in-person period, and (2) multiple shorter lock downs followed by multiple shorter in-person periods. The utility of this approach of having multiple strategies is that administrators of universities, schools, business, etc. can use a strategy that is best adapted for their own functionality.

## 1 Introduction

Coronavirus is the name given to a family of viruses with a characteristic spiked protein protrusion structure that resembles the solar corona [1]. In late 2019, a novel coronavirus named “severe acute respiratory syndrome coronavirus-2” (SARS-CoV-2), which causes the disease COVID-19, emerged in humans in Wuhan city, Hubei province, China [2]. Although its exact origins are unknown, evidence suggests that COVID-19 originated from zoonotic transfer via bats or pangolins with natural selection occurring either pre or post zoonotic transfer [3]. On March 11, 2020 the World Health Organization declared COVID-19 a global pandemic [4], where reports on death reached near 4 million worldwide by summer 2021 [5], and current (as of 05/2022) World Health Organization estimates suggest a total mortality of nearly 15 million [6]. With variants of the disease continuing to emerge, it is likely that the disease will persist into the foreseeable future, and models for understanding how to manage its spread are crucial.

Transmission of COVID-19 occurs in one of three ways: direct contact transmission, aerosol transmission, and droplet transmission [7]. Starting early in the COVID-19 pandemic, mitigation strategies such as masking, social distancing, lockdowns, and random testing were and are still being utilized to stop or slow down transmission of the disease [2, 8–10]. While vaccines with high efficacy have been developed, and have been shown to slow the virus spread [11], social behaviour and variant transmission have proven to be competing drivers of the virus, with newer variants such as “Delta” and “Omicron” spreading quickly through populations, especially those populations with low vaccination numbers [12]. In addition, children under 5, who are not cleared for vaccination in most of the world, and those vaccinated early on in the pandemic, are still transmitting the disease which has initiated new “booster” programs [13].

Previous models of COVID-19 spread have analyzed the dynamics of disease spread with the adherence and non-adherence of social behavior protocols such as masking, social distancing, and the enforcement of closures/lock downs [14–23]. To our knowledge, few models have incorporated the effect of randomized daily testing (although many universities have used this strategy to mitigate disease spread [24–26]) with the goal of maximizing in-person time utilizing feedback mechanisms while maintaining a low number of infections. This will be the focus of this work.

Here, we explore the dynamics of COVID-19 spread in a “closed” (e.g. self-contained) environment that incorporates testing, closures (i.e. lockdowns), and reopening. Examples of this type of system are universities with residential campuses that can be approximated as closed environments. The overall goal of this study is to provide insight to administrators interested in developing safe and effective reopening plans, based on our analyzed closure/reopen strategies, where we maximize the total in-person days while keeping infection numbers low. We do not explicitly take vaccination into account, as we assume all individuals in the system have the same vaccination status, which is a strategy taken by many US residential universities that require proof of vaccination upon arrival [27]. As we are interested in relatively short time frames (on the order of a single semester), we do not consider deferentially “boosted” population compartments, as this percentage of individuals is generally expected to be small. Furthermore, in environments with different vaccination requirements, model parameters can be re-calibrated, but our general modeling framework of a homogeneous population is valid. In addition, it has been shown that many vaccinated people can still contract and transmit certain variants of COVID-19 [13, 28].

We develop our model using an ordinary differential equation (ODE) modeling approach, based on extensions of the original Susceptible-Infected-Removed (SIR) model system developed by Kermack and McKendrick in 1927 [29]. The original SIR model includes compartments for susceptible individuals (S), infectious individuals (I), and those that have recovered or deceased (R). We extend this model and separate our infectious class into two categories: those that are asymptomatic but infectious (*I*_1_) and those that are symptomatic and infectious (*I*_2_). Note that we distinguish between these two classes since individuals who experience symptoms are more likely to practice social distancing and mask wearing, as well as avoid in-persons gatherings/classes. In addition to considering asymptomatic and symptomatic individuals, we also differentiate asymptomatic individuals into two sub-compartments: those that are unaware that they have contracted COVID-19 ($I_{1}^{u}$), and those that are aware via a positive test result ($I_{1}^{a}$).

In Section 2, we describe the model and its underlying assumptions in detail, as well as provide ranges for all model parameters. In addition, we provide an expression for the models basic reproductive number *R*_0_, based on a standard derivation of the model’s next generation matrix [30, 31], which we use to explore the dynamics of the model and to determine plausible values for the disease transmission rate. In Section 3, we explore the model behavior with respect to varying the reopening strategy. We keep the testing strategy fixed (exploring both a low and high testing rate), keep the closing criterion fixed, and identify optimal reopening strategies where reopening occurs when the percentage of the population remaining infected drops below a defined threshold (we vary this percentage between 0 and 5%).

## 2 Mathematical model

**Fig 1:**
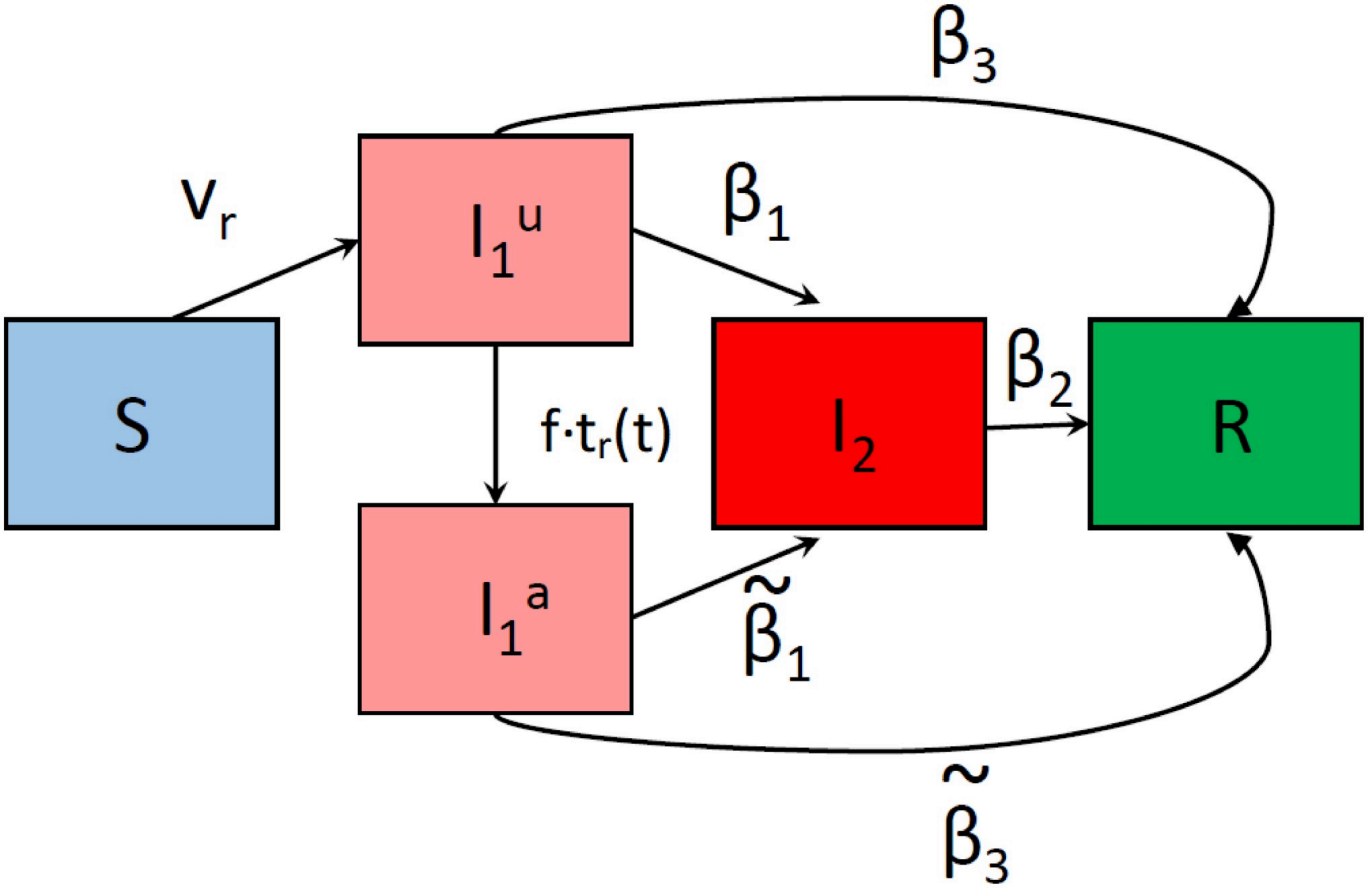
Compartmental diagram representing the transitions between different states in our model. Here, *v*_*r*_ describes the rate of acquiring COVID-19 (after an encounter with asymptomatic infectious individuals), *t*_*r*_(*t*) corresponds to the fraction of the population tested per day, where the parameter *f* is included to account for the fraction of true positive test results. The parameter *β*_1_ corresponds to the rate at which asymptomatic infectious individuals become symptomatic, *β*_2_ corresponds to the rate at which symptomatic individuals recover, and *β*_3_ corresponds to rate at which asymptomatic individuals recover (those that never become symptomatic). The “tildes” over *β*_1_ and *β*_3_ allow for the possibility of an increased rate of symptom development and recovery in $I_{1}^{a}$ individuals compared to $I_{1}^{u}$ individuals, simply because they are, on average, further along their disease progression having already spent time in the $I_{1}^{u}$ compartment.

Here, we introduce our ODE model of COVID-19 spread through a susceptible population, where initially a small percentage of the population is COVID-19 positive. This can be representative of a student body returning to campus after a break. Our model, described by Eqs (2) through (6), includes the following dependent variables: a susceptible population *S*, asymptomatic populations *I*_1_, which are separated into two classes, representing those that are aware that they have COVID-19 $I_{1}^{a}$, those that are unaware $I_{1}^{u}$, symptomatic infectious individuals *I*_2_, and individuals that are recovered/removed from the population *R*. Since we are considering a population of college students, we assume that removed individuals have recovered from the disease. We summarize how these populations interact with each other with a compartmental schematic of our model shown in Fig 1.

The schematic shows that susceptible individuals (*S*) become infected at a (transmission) rate *v*_*r*_, and are initially unaware they have COVID-19 ($I_{1}^{u}$). Either an individual becomes aware ($I_{1}^{a}$) that they have COVID-19 through testing (here *t*_*r*_(*t*) is the number of individuals tested per day and *f* is the fraction of true positive tests), or by becoming symptomatic (*I*_2_). Those individuals that show symptoms are considered COVID positive, and are required to quarantine until recovered. Individuals can recover (*R*) from either a symptomatic or asymptomatic state. Next, we describe the assumptions for which our model is developed.

### 2.1 Modeling assumptions

1. The rate of disease transmission, *v*_*r*_, depends on the number of contacts between a single susceptible individual and contagious asymptomatic infected individuals that are unaware they have COVID-19. This assumption is justified if we assume that people stick to the COVID-19 protocols strongly, and quarantine if they are aware they are infected through testing, or if they have symptoms.
2. We assume that the disease transmission rate *v*_*r*_ is primarily dependent on the number of close contacts (i.e., class sizes and number of individuals sharing a living space). Small numbers of students in a class lead to small transmission rates and larger numbers of students lead to larger transmission rates.
3. We assume testing is randomly performed (e.g. not targeted or performed via contact tracing) on the susceptible and asymptomatic infectious populations. Here *t*_*r*_(*t*) denotes the proportion of people tested (each day), and must generally be time-dependent, since we cannot test more than the total *testable population* $I_{1}^{u}+S$ at any time *t*. We assume a constant testing capacity *T*_*r*_, so that
  $$\begin{array}{cc} t_{r}\left(t\right) & =\text{min}\{T_{r},I_{1}^{u}\left(t\right)+S\left(t\right)\}. \end{array}$$ (1)
  Intuitively, we test a constant *t*_*r*_(*t*) = *T*_*r*_ individuals per day, unless the testable population falls below *T*_*r*_.
4. Testing is not perfectly accurate, which must be accounted for in our modeling framework. Parameter *f* represents the conditional probability of testing positive, given that the individual is infected.
5. The rate of transition between the asymptomatic and symptomatic states depends on testing, such that ${\tilde{\beta }}_{1}\leq \beta_{1}$. This accounts for the time lapse between getting infected and getting a positive test. Here we assume this time is negligible, so we set these values equal to each other.
6. In a similar light, the rate of transition between the asymptomatic and recovered states depends on testing, such that ${\tilde{\beta }}_{3}\leq \beta_{3}$.
7. Once infected individuals become aware that they are COVID-19 positive, they no longer infect susceptible individuals (i.e. they are quarantined).
8. We assume that recovered individuals are no longer infective or susceptible for the time scales considered in this work (generally a few months) [32].

### 2.2 The model equations

Eqs (2) through (6) describe the dynamics of COVID-19 through an initially susceptible population in the presence of testing. A small number of unaware infected individuals will be introduced into the system, as described by the initial condition for $I_{1}^{u}\left(0\right)$.

$$\begin{array}{cc} \frac{dS}{dt} & =-v_{r}(\frac{I_{1}^{u}}{I_{1}^{u}+S})S \end{array}$$ (2)

$$\begin{array}{cc} \frac{dI_{1}^{u}}{dt} & =v_{r}(\frac{I_{1}^{u}}{I_{1}^{u}+S})S-t_{r}\left(t\right)f(\frac{I_{1}^{u}}{I_{1}^{u}+S})-\left(\beta_{1}+\beta_{3}\right)I_{1}^{u} \end{array}$$ (3)

$$\begin{array}{cc} \frac{dI_{1}^{a}}{dt} & =t_{r}\left(t\right)f(\frac{I_{1}^{u}}{I_{1}^{u}+S})-\left({\tilde{\beta }}_{1}+{\tilde{\beta }}_{3}\right)I_{1}^{a} \end{array}$$ (4)

$$\begin{array}{cc} \frac{dI_{2}}{dt} & =\beta_{1}I_{1}^{u}+{\tilde{\beta }}_{1}I_{1}^{a}-\beta_{2}I_{2} \end{array}$$ (5)

$$\begin{array}{cc} \frac{dR}{dt} & =\beta_{2}I_{2}+\beta_{3}I_{1}^{u}+{\tilde{\beta }}_{3}I_{1}^{a} \end{array}$$ (6)

Eq (2) describes the rate at which susceptible individuals (*S*) become infected prior to exhibiting symptoms or testing positive, and are hence *unaware* they are infected. Parameter *v*_*r*_ is the rate of disease transmission. Since the testing is a random sample of the testable population, a test has a probability $I_{1}^{u}/\left(I_{1}^{u}+S\right)$ of being administered to a unaware infected individual. Eq (3) describes the rate of change of unaware infected individuals $I_{1}^{u}$, where the first term on the right-hand side describes disease transmission and the second term accounts for individuals becoming aware of their infection through testing. Rate *t*_*r*_(*t*) models random sampling of the *testable population*, which we assume is the sum of the susceptible population *S* and the unaware infected population $I_{1}^{u}$. Here *f* models the accuracy of the tests utilized, with *f* = 1 corresponding to a 100% accurate positive test. The third term on the right-hand side of (3) describes how unaware infected individuals either recover (at rate *β*_3_) or become symptomatic (at rate *β*_1_). Eq (4) describes the dynamics of *aware* asymptomatic individuals $I_{1}^{a}$, where the first term on the right-hand side accounts for positively tested asymptomatic individuals. The second term accounts for an asymptomatic individual either becoming symptomatic (at rate ${\tilde{\beta }}_{1}$) or recovered (at rate ${\tilde{\beta }}_{3}$). Eq (5) describes the dynamics of symptomatic individuals *I*_2_. Here, the first two terms on the right-hand side account for individuals developing symptoms from either being unaware (at rate *β*_1_) or aware (at rate ${\tilde{\beta }}_{1}$) asymptomatic individuals. Note that in our model, the asymptomatic compartment includes both individuals that will never develop symptoms, as well as individuals that are currently asymptomatic but will later go on to develop symptoms (often referred to as ‘pre-symptomatic’). The final term in this equation describes the transition into a recovered state *R*, which occurs at rate *β*_2_. The final Eq (6) corresponds to recovery from either asymptomatic or symptomatic states. All model variables and parameters, along with the range of values used in this paper, are summarized in Table 1 and are discussed more fully in Section 2.4.

**Table 1:** Table of parameter descriptions and ranges of values used in the model. All parameters are assumed non-negative. *S*(0), $I_{1}^{u}\left(0\right)$, $I_{1}^{a}\left(0\right)$, *I*_2_(0), and *R*(0) define the initial population sizes. Dashes are used when values are arbitrarily chosen from some range.

Parameter	Description	Range of Values	References
*T*_*r*_	testing capacity	[0, 1] (*day*^−1^)	-
*v*_*r*_	transmission rate	[0, 1] (*day*^−1^)	[35–41]
*f*	fraction of true positives	[0.815, 0.922]	[42]
*β*_1_	Incubation rate (from unaware)	[0.143, 0.224] (*day*^−1^)	[43–45]
*β*_2_	Rate of recovery (from symptomatic)	[0.05, 0.1] (*day*^−1^)	[46]
*β*_3_	Rate of recovery (from unaware)	[0.04, 0.07] (*day*^−1^)	-
*S*(0)	Initial fraction of susceptible individuals	1 − *r*, *r* = 0.01	-
$I_{1}^{u}\left(0\right)$	Initial fraction of unaware individuals	*r*, where *r* ≪1	-
$I_{1}^{a}\left(0\right)$	Initial fraction of aware individuals	0	-
*I*_2_(0)	Initial fraction of symptomatic individuals	0	-
*R*(0)	Initial fraction of recovered individuals	0	-

Here, we are assuming that once individuals recover, they are “removed” from the system such that they can no longer be infected again, although there is evidence that some individuals can contract COVID-19 a second time (although typically at longer times after the first infection than those times investigated here) [33]. Current work is being completed to determine how often reinfection does occur, and how long after infection can reinfection occur [33]. With the possibility of reinfection, and an ever increasing number of variants of COVID-19 including the more transmissible *Delta* and *Omicron* variants which are transmissible even to vaccinated individuals [34], future work may lead to incorporating re-entry back into susceptible populations.

By the structure of system (2)–(6), it is clear that dynamics are positively invariant, and furthermore, that the total population

$$\begin{array}{c} N(t)\;≔\;S\left(t\right)+I_{1}^{u}\left(t\right)+I_{1}^{a}\left(t\right)+I_{2}\left(t\right)+R\left(t\right) \end{array}$$ (7)

is a conserved quantity, i.e. *N*(*t*)≡*N*(0). Hence, for the remainder of this work, we normalize all model variables with respect to the initial (and thus total) population such that

$$\begin{array}{cc} N(t) & \equiv 1. \end{array}$$ (8)

This then implies that rate parameters should be interpreted with respect to population fractions, as opposed to raw population numbers. For example, testing capacity *T*_*r*_ thus represents the *proportion* of the testable population that can be tested per day.

### 2.3 Calculating the basic reproductive number *R*_0_

Two important components in understanding any disease are (1) to determine the mechanisms by which a disease spreads through a population, and (2) to determine strategies that increase the chances of stopping a disease outbreak. Mathematical epidemiologists work to address these issues is various ways, one being to determine the stability of the models disease-free equilibrium/equilibria (DFE), which can often be found by determining an expression for the basic reproductive number *R*_0_, defined as the expected number of infected individuals that results from a single infected individual being placed into a completely susceptible population [30]. According to Diekmann et al. [31], the *R*_0_ can be calculated as the spectral radius of a Next-Generation Matrix (NGM). The calculation of *R*_0_ from the NGM is standard, and we refer the reader to [30, 31] for further details of the calculation. Here, we find *R*_0_ to be

$$\begin{array}{c} R_{0}=\frac{v_{r}{\left(S^{*}\right)}^{2}}{t_{r}\left(t\right)f+S^{*}\left(\beta_{1}+\beta_{3}\right)}, \end{array}$$ (9)

where S* corresponds to the models susceptible population at equilibrium. We observe that larger disease transmission rates *v*_*r*_ and lower testing capacity *t*_*r*_(*t*) result in higher values of *R*_0_, such that a disease outbreak is more likely. In the Results Section we illustrate two possible outcomes: *R*_0_ > 1 corresponding to disease persistence, and *R*_0_ < 1 corresponding to disease die out.

### 2.4 Parameter estimation

Table 1 summarizes all variables and parameters for the model described in Eqs (2) through (6). In particular we provide the meaning of each parameter, as well as providing a full description for how each model parameter was chosen (either from mathematical constraints, or inferred from the literature). All parameter values were determined using data from the ancestral COVID-19 strain. However, as explained later in the Results Section, our model is robust to changes in certain parameters, including the disease transmission rate *v*_*r*_ (exceptions include the disease incubation rate *β*_1_ and the recovery after symptoms rate *β*_2_, parameters that are relatively easy to determine from clinical data). Since it can be difficult to determine the transmission rate of emerging COVID-19 variants, and even the original COVID-19 strain, our model is easily extended to address questions about the spread of the newer variants, and will likely show negligible difference in the optimal reopening strategies when compared to our results.

***v*_*r*_: Transmission rate**. To calculate the transmission rate *v*_*r*_ we use Eq 9. That is, for a chosen *R*_0_ value and input values for S*, *t*_*r*_(*t*), *f*, *β*_1_, and *β*_3_, we calculate *v*_*r*_.

***T*_*r*_: Testing capacity**. The testing capacity corresponds to the proportion of the testable population that *can be* tested (and returned) per day. Generally the tested population is the constant *T*_*r*_, unless in the rare case that the testable population becomes too small (see Eq 1). Hence *T*_*r*_ ∈ [0, 1]. Two types of tests are currently available: viral tests and antibody tests. A viral test tells you if you have the infection, and an antibody test can tell you if you had the infection. Here, we focus on the former. The two major types of viral tests, nucleic acid and antigen tests, have different return times. Antigen tests (typically referred to as “rapid testing”) are faster with a turnaround time of 15 minutes. Nucleic acid tests (PCR testing) may take between 15 minutes to over 2 days [47]. For simplicity, we assume that all tests performed are PCR tests which are returned within 24 hours.

***f*: Fraction of true positives**. Parameter *f* represents the probability of testing positive, given that the tested individual is infected. Molecular tests are more accurate than antigen tests. Specifically, the sensitivity (fraction of true positives) of molecular tests using PCR is estimated to be 87.8% (95% CI: 81.5% to 92.2%) [42] compared with antigen tests which have a mean sensitivity of 58% for asymptomatic people [48]. Consequently, we assume 0.815 ≤ *f* ≤ 0.922.

**Transition rates *β*_*i*_** (**and** $\tilde{\beta_{i}}$). All transition rates *β*_*i*_ and $\tilde{\beta_{i}}$ are interpreted as the expected value of a Poisson process. Specifically, we can relate each *β*_*i*_ to the expected transition time 〈*t*_*s*_〉 via

$$\begin{array}{c} \beta_{i}=\frac{1}{\left\langle t_{s}\right\rangle }. \end{array}$$ (10)

For example, the rate of developing symptoms *β*_1_ is the reciprocal of the time spent asymptomatic (given that the individual did not test positive or recover).

***β*_1_** (**and** $\tilde{\beta_{1}}$): **Incubation rate**. Incubation periods typically vary from one study to the other, due to differences in study methods and populations. An early study of 425 confirmed cases in the Hubei province in Wuhan found the mean incubation period to be 5.2 days with a 95% confidence interval of 4.1 to 7.0 days [43]. A recent meta analysis of reported incubation period from January 2020 to May 2021 found the 95% confidence interval to be [5.70, 6.45] [49]. Another meta analysis of 99 studies found the 95% confidence interval for the incubation period to be [5.79, 6.97] [50]. All these intervals lie within range [4.1, 7.0] used in this study. We estimate the incubation rate *β*_1_ to range from 0.143 to 0.224 (day^−1^). ${\tilde{\beta }}_{1}$ corresponds to the incubation rate after the individual has received a positive test, which may be faster than *β*_1_ depending on how much time the individual spends infected before receiving that positive test result. We simplify the model by assuming this difference is negligible and set ${\tilde{\beta }}_{1}\;=\beta_{1}$.

***β*_2_: Recovery rate of symptomatic individuals**. The Centers for Disease Control and Prevention recommends ending isolation for people infected with mild cases of SARS-CoV-2 after at least 10 days since onset of symptoms and up to 20 days if the illness is severe [46]. Based on these numbers, we estimated the recovery time after symptom onset to range between 10 and 20 days to cover both severe and mild illness. Our estimated recovery rate from symptomatic state, *β*_2_ thus ranges from 0.05 − 0.1 day^−1^

***β*_3_** (**and** $\tilde{\beta_{3}}$): **Rate of recovery from asymptomatic individuals**. To estimate the recovery time from asymptomatic state we shifted the recovery time from symptomatic state forward by the mean incubation period of 5.2 days. Thus, our estimated range for recovery rate from asymptomatic state, *β*_3_ is 0.04 − 0.07 day^−1^. ${\tilde{\beta }}_{3}$ is the rate of recovery from the aware asymptomatic state. Following the same reasoning outlined above for ${\tilde{\beta }}_{1}$, we set ${\tilde{\beta }}_{3}=\beta_{3}$.

### 2.5 Methods

We assume that our population represents a small residential college environment, where we require the total infected population to remain below a fraction *p* of the total population, during an academic semester. Furthermore, as administrators of such colleges only have information about COVID aware (those whom tested positive) and symptomatic individuals, we work to understand the dynamics of our model with respect to the following metric:

$$\begin{array}{c} t_{open}\;≔\;\text{sup}\{T\geq 0\;|\;\tilde{p}N\leq I_{1}^{a}\left(t\right)+I_{2}\left(t\right)\leq pN,\forall t\in \left[0,T\right]\}. \end{array}$$ (11)

The metric *t*_*open*_ represents the total in-person time spent at an institution, where we assume that after a closure (when a proportion *p* of the population becomes infected) the institution will reopen when the remaining proportion of the population who is infected drops below $\tilde{p}$. We assume that the institution will then move back to in-person status until the proportion of infected individuals again reaches *p*, and another closure occurs.

Note that we will assume that $I_{1}^{a}\left(0\right)+I_{2}\left(0\right)<pN$, so that *t*_*open*_ > 0. Furthermore, the supremum in (11) is necessary (as opposed to a maximum), as disease/control parameters may be such that $I_{1}^{a}\left(t\right)+I_{2}\left(t\right)\leq pN$ for all *t* ≥ 0.

As noted in (8), without loss of generality we fix *N* = 1 in (11). Lastly, since $I_{1}^{a}\left(t\right)+I_{2}\left(t\right)\leq pN$ for all times *t* ∈ [0, *T*] if and only if

$$\begin{array}{cc} \underset{t\in [0,T]}{\text{max}}\left\{I_{1}^{a}\left(t\right)+I_{2}\left(t\right)\right\} & \leq pN, \end{array}$$

For definiteness, we fix

$$\begin{array}{c} p=0.05. \end{array}$$ (12)

This value of *p* is based on protocols utilized at different institutions throughout the USA, and in particular those used in New York state in Spring and Fall 2020 semesters [51]. Further, we define

$$\begin{array}{c} 0<\tilde{p}<0.05. \end{array}$$ (13)

In recent semesters, the original SARS-CoV-2 strain has evolved into multiple variants of concern, including the *Delta* and *Omicron* variants [12]. Even those individuals that have been fully vaccinated (and boosted) have been able to contract and transmit the virus [52]. As such, institutions must consider new strategies that can provide students with an optimal face to face experience, all while keeping them safe. Thus, understanding and maximizing the in-person time *t*_*open*_ with respect to $\tilde{p}$ (the proportion of the population infected at reopening) is crucial.

We look at optimal reopening strategies for universities based on infection numbers, where we look to optimize in-person class time with respect to varying infected population sizes, $\tilde{p}$ of the total population, at the time of reopening. We consider a closure to occur when the infection numbers reach 5% of the total population (i.e., when *p* = 0.05) and consider reopening when the infection numbers drop to a proportion $\tilde{p}$ of the entire population (here $0<\tilde{p}<0.05$). Furthermore, if the percentage of the infected population reaches 5% again, we consider another closure (and so on).

## 3 Results

Here we simulate our model given by Eqs (2) through (6) under a base case set of parameters (found in Table 1) to highlight the dynamics of disease spread in two cases (i.e., disease die out when *R*_0_ < 1 and disease spread when *R*_0_ > 1). Then, we illustrate how variations in the disease transmission rate *v*_*r*_ lead to different reopening strategies. As stated previously, a closure occurs when the proportion of infected people reaches *p* = 0.05 and we explore the optimal reopening strategies by examining the maximum number of in-person days *t*_*open*_ as the proportion of infecteds at reopening, $\tilde{p}$, is varied (between 0 and 0.05).

### 3.1 Baseline model dynamics: Disease outbreak vs disease die out

**Fig 2:**
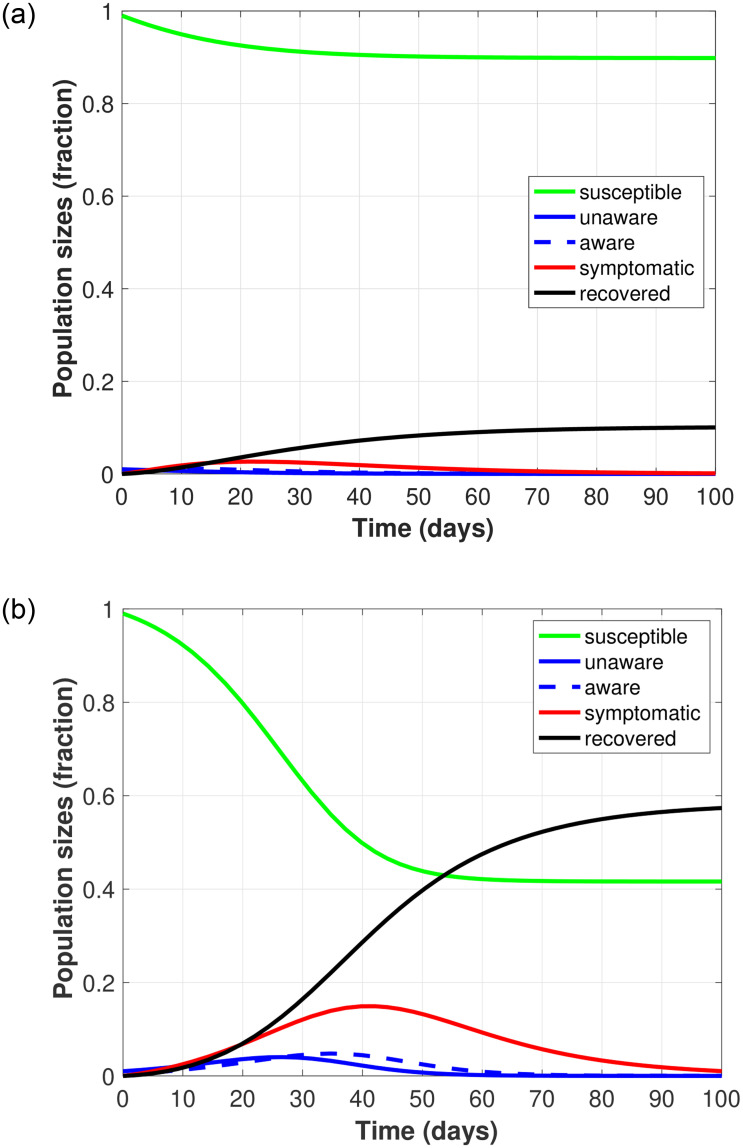
Illustration of the progression of COVID-19 over a 100 day time period. (a) *R*_0_ < 1 indicates that the disease dies out when *v*_*r*_ = 0.5 and *T*_*r*_ = 0.4, and (b) *R*_0_ > 1 indicates local stability of the disease-free equilibria set when *v*_*r*_ = 0.45 and *T*_*r*_ = 0.2. Here, a portion of individuals from the testable population ($S+I_{1}^{u}$) are tested every day.

Fig 2 corresponds to the two disease progression possibilities based on the value of the basic reproductive number *R*_0_ for two different pairs of parameters *v*_*r*_ and *T*_*r*_ (transmission rate and testing capacity) and for the baseline parameter set given in Table 2. That is, we illustrate disease die out when *R*_0_ < 1 (Fig 2(a)) and disease outbreak when *R*_0_ > 1 (Fig 2(b)).

**Table 2:** Base set of parameters and initial conditions for our model. We assume that 1% of the initial population is infected and are unaware of their infection.

Baseline Parameters and Initial Conditions										
*S*(0)	$I_{1}^{u}\left(0\right)$	$I_{1}^{a}\left(0\right)$	*I*_2_(0)	*R*(0)	*f*	*β*_1_ (t -1)	*β*_2_ (t -1)	*β*_3_ (t -1)	$\tilde{\beta_{1}}$ (t -1)	$\tilde{\beta_{3}}$ (t -1)
0.99	0.01	0	0	0	0.85	0.143	0.06	0.05	0.143	0.05

In Fig 2(a) we see that the disease dies out quickly (shown by the small rise and fall of the blue and red curves), and only a small proportion of the susceptible population becomes infected (shown by the green curve). In Fig 2(b) we see that the disease persists for longer, shown by the large peak in symptomatic infecteds (shown by the red curve). Here, approximately 60% of the susceptible population becomes infected (shown by the green curve), before eventually recovering (shown by the black curve).

It should be noted that our model performed similarly in the case where we considered imperfect quarantine. That is, when we incorporated a term in our model to account for a small number of symptomatic individuals transmitting the disease to susceptible individuals, we recovered model dynamics very similar to our baseline dynamics described in Fig 2 (results not shown). As we assume that individuals follow strict quarantine in our modeling framework, we expect imperfect quarantine to be negligible here.

### 3.2 Reopening strategies for variations in the disease transmission rate *v*_*r*_

One valuable result of our model is that the reopening strategies are robust to variations in the transmission rate *v*_*r*_ when *v*_*r*_ is large in value and the testing capacity *T*_*r*_ is fixed. The transmission rate can vary widely depending on variant of the virus and is likely the parameter that is most difficult to calculate. In fact, our model is robust to all model parameters except *β*_1_ (the incubation rate) and *β*_2_ (the rate of recovery), both of which are quite straightforward to calculate. Here, robustness to our choice of most model parameters means that, for a given testing strategy *T*_*r*_, the optimal reopening strategies remain similar. In order to show the robustness of our model to all parameters, we plot histograms that bin the number of occurrences of the optimal $\tilde{p}$ values that fall within 5% of the maxima for *t*_*open*_. In what follows we highlight the robustness of the transmission rate *v*_*r*_ parameter choice and include a description of robustness for all other model parameters in S1 File.

**Fig 3:**
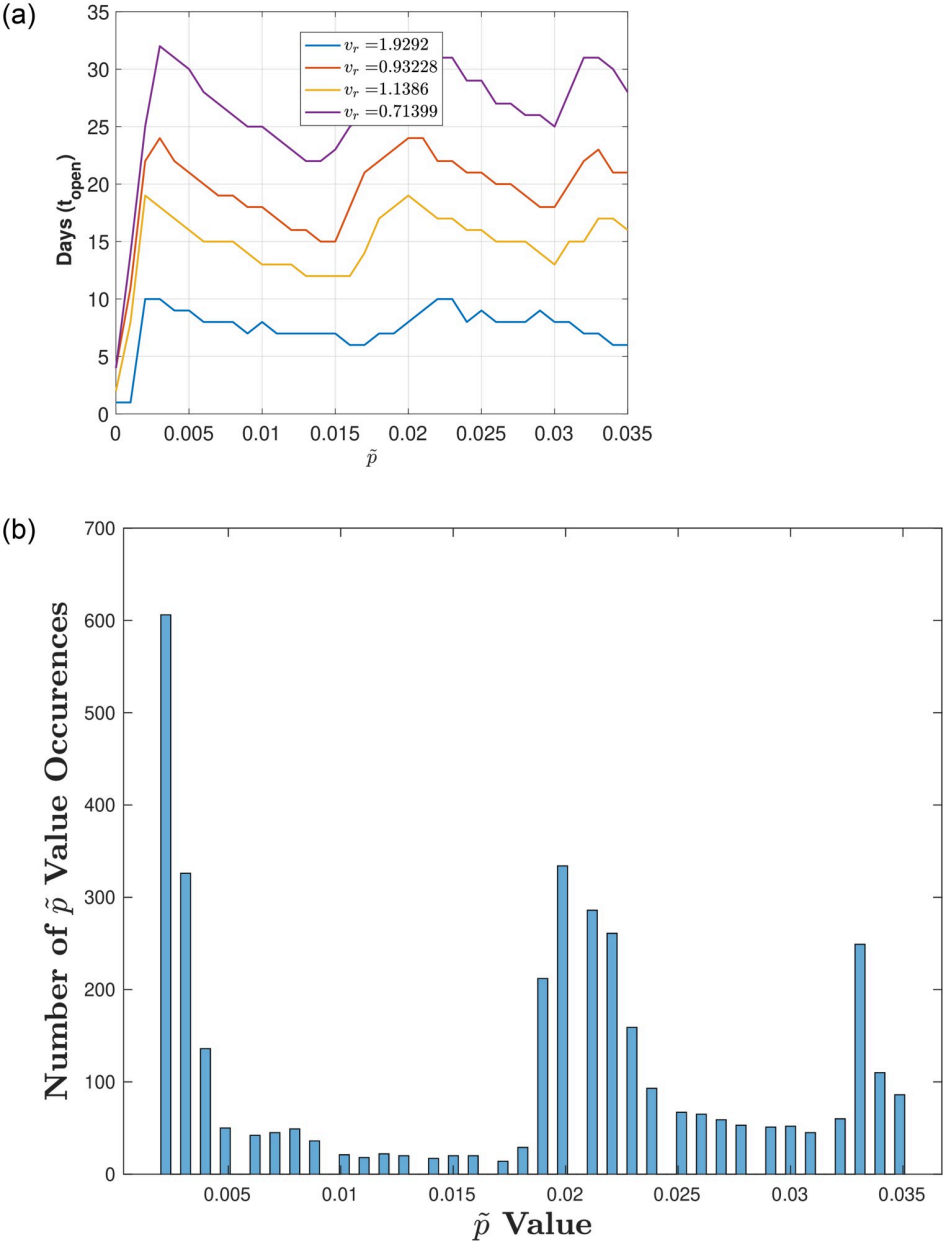
Exploring reopening strategies for a wide range in disease transmission *v*_*r*_ with testing capacity. *T*_*r*_ = 0.1: We randomly choose *v*_*r*_ from a wide range of values (0.3 to 2 which corresponds to *R*_0_ values between 1.1 and 7.1). (a) Plot of in-person days *t*_*open*_ for a varying reopening proportion of infecteds $\tilde{p}$. (b) Histogram plot of the $\tilde{p}$ values that result in a number of in-person days that are within 5% of the maximum (defined by the peaks in (a)). Here, 75 bins are used and 1000 values of *v*_*r*_ are uniformly chosen between 0.3 and 2.

**Fig 4:**
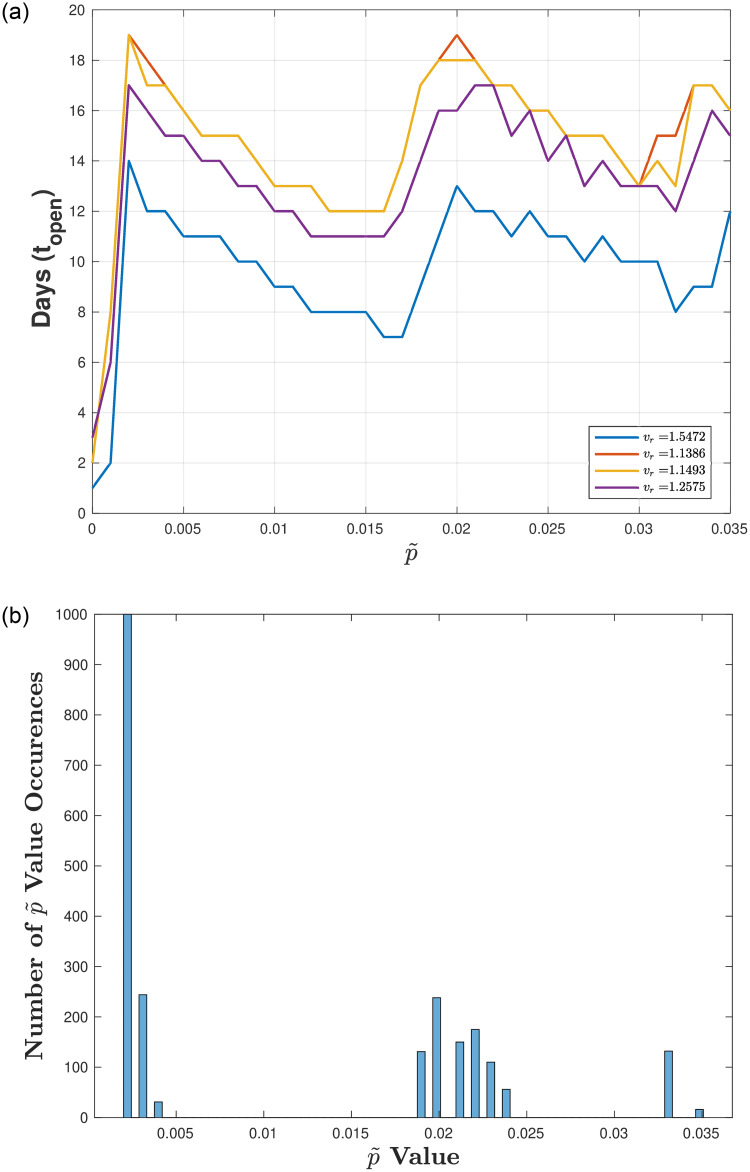
Exploring reopening strategies for high disease transmission *v*_*r*_ with testing capacity. *T*_*r*_ = 0.1: We randomly choose *v*_*r*_ from a range of values (1 to 2 which corresponds to *R*_0_ values between 3.6 and 7.1). (a) Plot of in-person days *t*_*open*_ for a varying reopening proportion of infecteds $\tilde{p}$. (b) Histogram plot of the $\tilde{p}$ values that result in a number of in-person days that are within 5% of the maximum (defined by the peaks in (a)). Here, 75 bins are used and 1000 values of *v*_*r*_ are uniformly chosen between 1 and 2.

**Fig 5:**
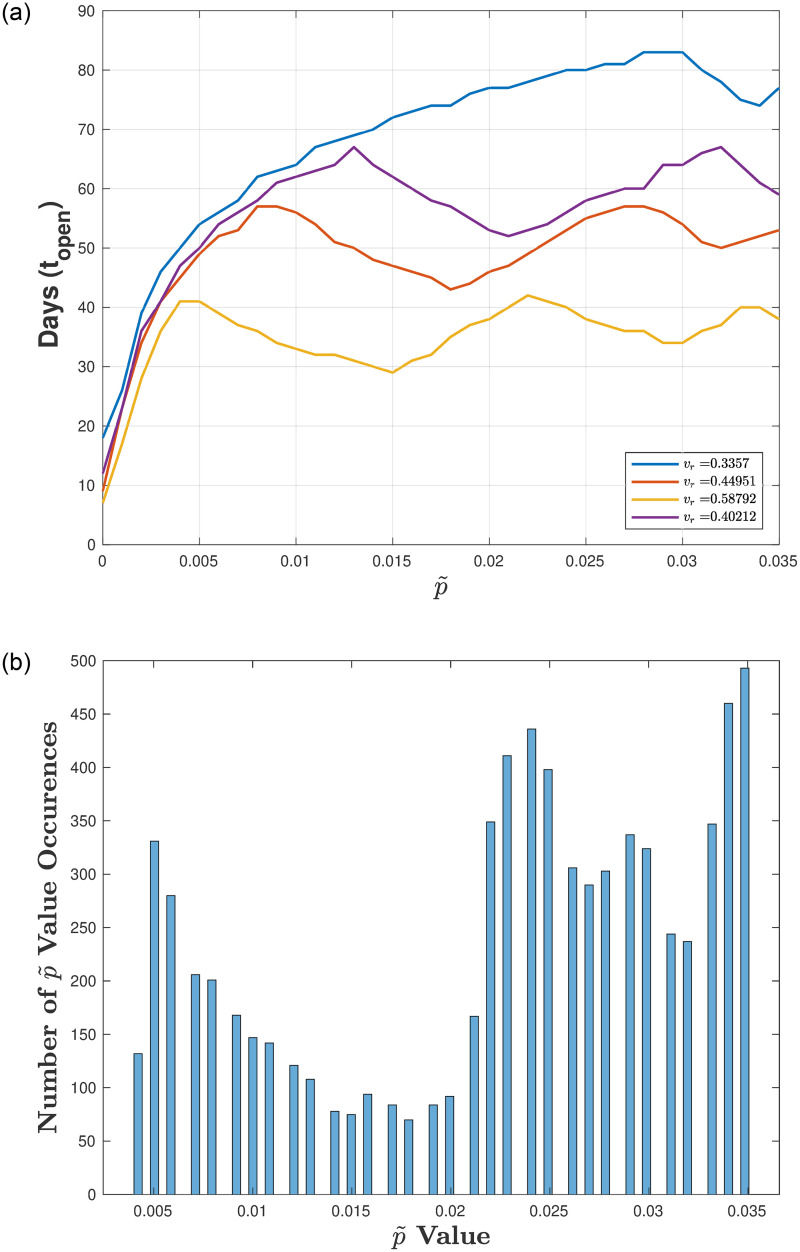
Exploring reopening strategies for low disease transmission *v*_*r*_ with testing capacity. *T*_*r*_ = 0.1: We randomly choose *v*_*r*_ from a range of values (0.3 to 0.6 which corresponds to *R*_0_ values between 1.1 and 2.1). (a) Plot of in-person days *t*_*open*_ for a varying reopening proportion of infecteds $\tilde{p}$. (b) Histogram plot of the $\tilde{p}$ values that result in a number of in-person days that are within 5% of the maximum (defined by the peaks in (a)). Here, 75 bins are used and 1000 values of *v*_*r*_ are uniformly chosen between 0.3 and 0.6. Notice here that the peaks are not well defined in the sense that each *v*_*r*_ has different optimal *t*_*open*_.

Figs 3–5 illustrate the optimal number of in-person days *t*_*open*_ for varying $\tilde{p}$ (the proportion of infecteds at reopening) for a fixed testing strategy (*T*_*r*_ = 0.1) and different ranges of the transmission rate *v*_*r*_. Fig 3 corresponds to a full range of transmission rates (*v*_*r*_ low to high), Fig 4 corresponds to a high range of transmission rates, and Fig 5 corresponds to a low range of transmission rates.

Fig 3 illustrates results for low and high transition rates *v*_*r*_ (values between 0.3 and 2), where Fig 3(a) shows 3 peaks for *t*_*open*_ as we vary $\tilde{p}$ from 0 to 0.035 (there are no additional peaks after 0.035—results not shown). To determine whether these $\tilde{p}$ values indeed result in peaks (i.e., optimal *t*_*open*_) we plot a histogram in Fig 3(b) that bins the number of occurrences of the optimal $\tilde{p}$ values that fall within 5% of the maxima for *t*_*open*_. Although we do see peaks in $\tilde{p}$, there is a spread in $\tilde{p}$ values, and the distinction between peaks/optimal values of *t*_*open*_ is greater for larger *v*_*r*_ and smaller for *v*_*r*_ small. To illustrate this, we separate the results in terms of low values of *v*_*r*_ and high values of *v*_*r*_.

Fig 4(a) illustrates that for high *v*_*r*_ (values between 1 and 2) there are 3 distinct peaks for *t*_*open*_ that are considerably higher than their corresponding minima as we vary $\tilde{p}$ from 0 to 0.035 (there are no additional peaks after 0.035—results not shown). To determine whether these values are indeed peaks, we again plot a histogram that bins the number of occurrences of the optimal $\tilde{p}$ values that fall within 5% of *t*_*open*_. In Fig 4(b) we see 3 distinct peaks for varying $\tilde{p}$, corresponding to the optimal *t*_*open*_.

Fig 5(a) shows that for low ranges of *v*_*r*_ (values between 0.3 and 0.6), no clear peaks amongst the *v*_*r*_ values exist as we vary $\tilde{p}$. Furthermore, Fig 5(b) illustrates that the histogram that bins the number of occurrences of the optimal $\tilde{p}$ values shows no clear distinctions between the $\tilde{p}$ values. This result suggests that for low enough *v*_*r*_ values, variations in the opening strategy, dictated by the choice in $\tilde{p}$, does not result in significant differences. Therefore, we conclude that for high *v*_*r*_ values there are 3 distinct optimal strategies and that for low *v*_*r*_ values different reopening strategies do not result in significant differences. We note that situations in which the disease transmission *v*_*r*_ is low do not often cause significant shutdowns and as such are not as crucial to study in terms of reopening strategies.

**Fig 6:**
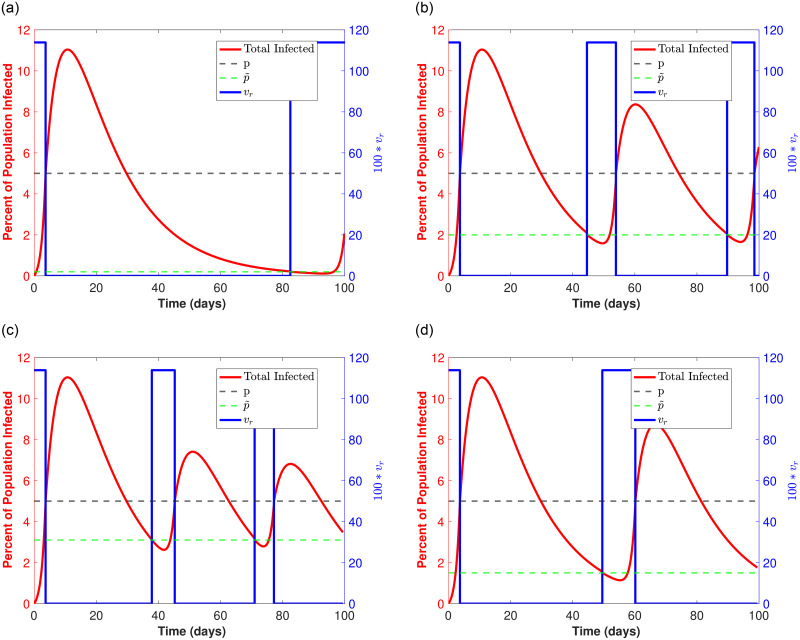
Optimal strategies for high *v*_*R*_ = 1.1386 and *T*_*R*_ = 0.1: (10% of population tested daily). The red curve corresponds to the infected population over time, and the *v*_*r*_ shifts from high to zero to signify *open* and *closed* periods, respectively (*v*_*r*_ is the blue step function). Note that as soon as the red curve reaches 5% (black dotted horizontal line), there is a closure and *v*_*r*_ = 0. The population of infecteds continues to rise as there are infections still working through the population, but eventually drops (staying below 10% in all cases). When infecteds drop to the indicated $\tilde{p}$ (green dotted horizontal line) there is a reopening and *v*_*r*_ = 1.1386 again. The process repeats over a time span of *T* = 100 days. **(a)** $\tilde{p}=0.002$. **(b)** $\tilde{p}=0.02$. **(c)** $\tilde{p}=0.031$. **(d)** $\tilde{p}=0.015$.

To better highlight the three optimal strategies described in Fig 4 (when *v*_*r*_ is high) we set the transmission rate *v*_*r*_ = 1.1386 (corresponding to the red curve in Fig 4(a)) and plot the curves for the percent infected population over time for each of the optimal reopening $\tilde{p}$ values in Fig 6. Here, the blue switch function corresponds to periods of time when things are open such that the transmission rate *v*_*r*_ = 1.1386 and when things are closed such that *v*_*r*_ = 0. Also, the black and green dotted lines correspond to the proportion of infecteds when a closure occurs (*p* = 0.05) and the proportion of infecteds upon reopening (the optimal $\tilde{p}$), respectively.

Fig 6(a)–6(c), correspond to the first, second, and third optimal strategy illustrated by the peaks in Fig 4(a) (red curve), respectively. In each case the maximum number of in-person days *t*_*open*_ is just over 20 days, where Fig 6(a) describes a single long closure after less than a week open, followed by approximately two more in-person weeks at the semester’s end. Fig 6(b) shows roughly 2 shorter closures after less than an initial week open. Here, we have two 10 day in-person periods in between those closures. Finally, Fig 6(c) shows 3 even shorter closures after the first closure, where there are 2 week long (roughly) in-person time periods.

To illustrate a *bad* strategy, we also plot results corresponding to a minimum of in-person days *t*_*open*_ with $\tilde{p}$ = 0.015 in Fig 6(d). This minimum is shown in Fig 4(a) (first minimum after the first maximum along purple curve). Here we see two extended closures after the initial closure, with a single in-person time period of 10 days. In total there are under 15 days of in-person time as opposed to the optimal (more than) 20 in-person days that we have for the 3 optimal strategies.

### 3.3 Reopening strategies for high testing capacity *T*_*r*_

**Fig 7:**
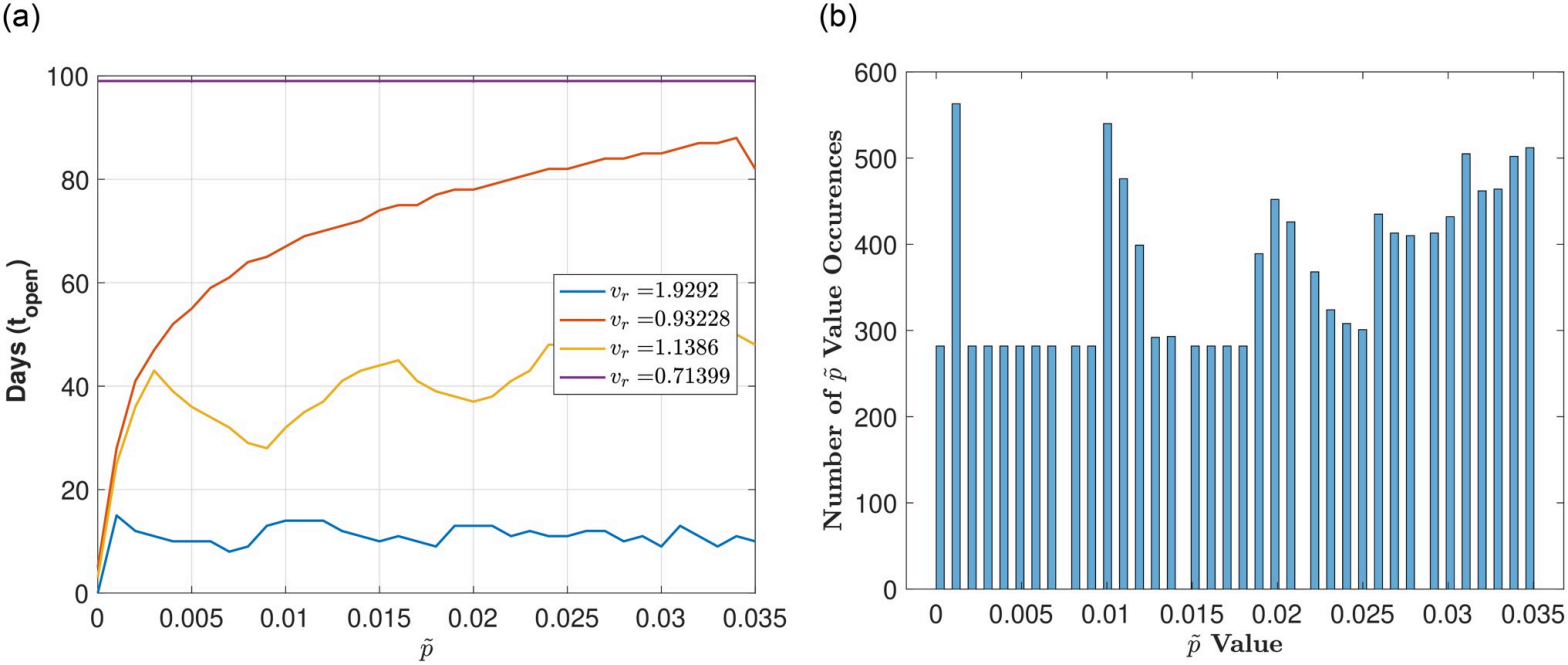
Exploring reopening strategies for a wide range in disease transmission *v*_*r*_ with high testing capacity *T*_*r*_ = 0.75: We randomly choose *v*_*r*_ from a range of values (0.3 to 2) which corresponds to *R*_0_ values between 1.1 and 7.1). (a) Plot of in-person days *t*_*open*_ for a varying reopening proportion of infecteds $\tilde{p}$. (b) Histogram plot of the $\tilde{p}$ values that result in a number of in-person days that are within 5% of the maximum (defined by the peaks in (a)). Here, 75 bins are used and 1000 values of *v*_*r*_ are uniformly chosen between 0.3 and 2. Notice here that the peaks are not well defined in the sense that each *v*_*r*_ has different optimal *t*_*open*_.

**Fig 8:**
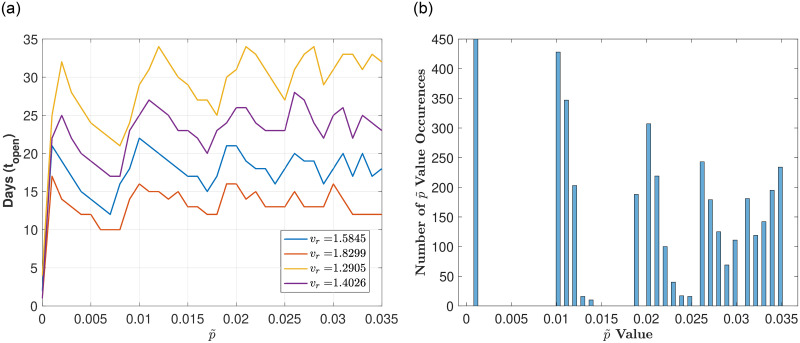
Exploring reopening strategies for a high range in disease transmission *v*_*r*_ with high testing capacity. *T*_*r*_ = 0.75: We randomly choose *v*_*r*_ from a range of values (1 to 2) which corresponds to *R*_0_ values between 3.6 and 7.1). (a) Plot of in-person days *t*_*open*_ for a varying reopening proportion of infecteds $\tilde{p}$. (b) Histogram plot of the $\tilde{p}$ values that result in a number of in-person days that are within 5% of the maximum (defined by the peaks in (a)). Here, Here, 75 bins are used and 1000 values of *v*_*r*_ are uniformly chosen between 1 and 2. Notice here that the peaks are well defined in the sense that each *v*_*r*_ has similar optimal *t*_*open*_.

Finally, we test how our optimal reopening strategies change if we increase our testing capacity *T*_*r*_, where all previous work in this section has fixed *T*_*r*_ = 0.1. By increasing *T*_*r*_, we see that longer in-person time periods exist, as expected. Fig 7 illustrates results for low and high transition rates *v*_*r*_ (values between 0.3 and 2), where Fig 7(a) shows a large variation is opening strategies as we vary *v*_*r*_ from low to high (i.e., distinct peaks are not obvious). In addition, we see that for *v*_*r*_ low enough, no closures exists such that *t*_*open*_ = 100 days (shown by purple curve). Fig 7(b) shows the histogram plot of the $\tilde{p}$ values that result in a number of in-person days that are within 5% of the maxima, highlighting that no distinct strategies exist when examining low to high *v*_*r*_ together. As in the case when *T*_*r*_ was low, *v*_*r*_ is robust to changes in $\tilde{p}$ only when it is high (*v*_*r*_ in range 1 to 2). Fig 8(a) and 8(b) illustrate that two peaks exist when $\tilde{p}$ is low ($\tilde{p}<0.015$), and that there are no clearly defined reopening strategies when $\tilde{p}$ is higher (peaks are less well defined).

**Fig 9:**
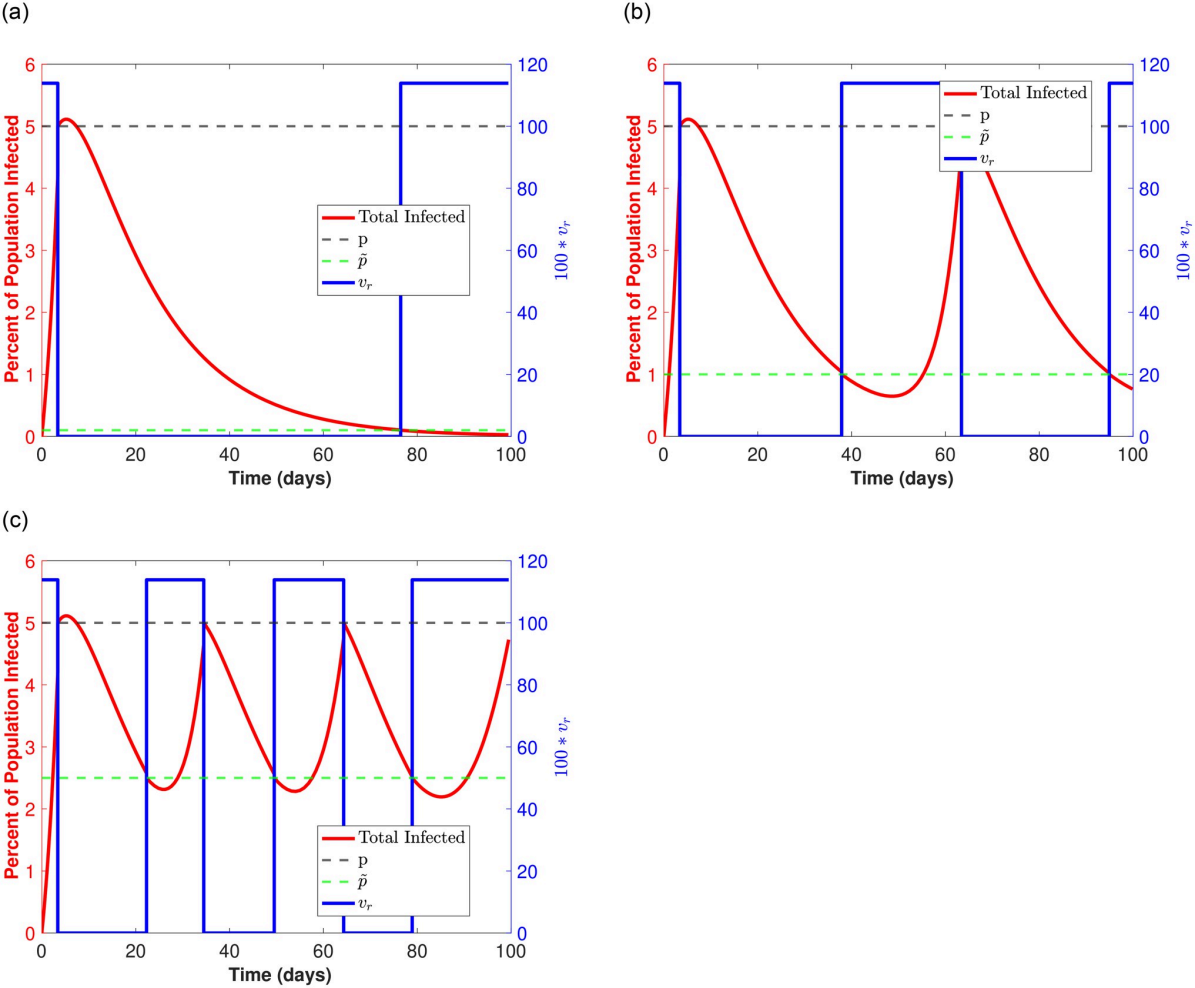
Optimal strategies for high *v*_*R*_ = 1.1386 and high *T*_*R*_ = 0.75: (75% of population tested daily). The red curve corresponds to the infected population over time, and the *v*_*r*_ shifts from high to zero to signify *open* and *closed* time periods, respectively (*v*_*r*_ is the blue step function). Note that as soon as the red curve reaches 5% (black dotted horizontal line), there is a closure and *v*_*r*_ = 0. The population of infecteds only rise slightly above 5% (in all cases) and eventually drop. When infecteds drop to the indicated $\tilde{p}$ (green dotted horizontal line) there is a reopening and *v*_*r*_ = 1.1386 again. The process repeats over a time span of *T* = 100 days. **(a)** $\tilde{p}=0.001$. **(b)** $\tilde{p}=0.01$. **(c)** $\tilde{p}=0.025$.

We compare our previous testing strategies for *T*_*r*_ = 0.1 using the same model parameters given in Table 1, changing *T*_*r*_ = 0.75 and examining the reopening strategies when *v*_*r*_ = 1.2 (optimal strategies highlighted by peaks in the yellow curve in Fig 8). We illustrate the different reopening strategies that allow for the longest in-person time, *t*_*open*_, in Fig 9(a) and 9(b) which correspond to the first and second optimal strategy when $\tilde{p}<0.015$ illustrated by the first 2 peaks in Fig 8(a) and 8(b) (yellow curve for *v*_*r*_ = 1.2). In addition we choose an additional strategy for $\tilde{p}>0.015$ in Figure (c). The maximum in-person time remains between 30 and 35 days. In Fig 9(a) we see an extended break after the first shut down followed by a single lengthy in-person end of semester. In Fig 9(b) we have two shorter closures after the first shut down, each followed by two in-person sessions, and in Fig 9(c) we see three short shut downs (between 10 and 14 days), followed by 3 in-person sessions (all greater than 10 days).

## 4 Conclusions and discussion

Here we developed and analysed a model of COVID spread in a closed environment (like a residential-campus university) that takes into account weekly testing as well as reopening strategies. The overall goal of the study was to optimize the number of in-person days (versus virtual) for varying reopening strategies. We explored the possibility of reopening after closing when a proportion *p* = 0.05 of the population becomes infected, where reopening is based on the proportion $\tilde{p}$ of people still infected ($\tilde{p}<p$). With variants of the disease emerging rapidly, and with individuals still contracting and transmitting the virus when vaccinated, it is likely that the disease will persist into the foreseeable future. Thus, models for understanding how we can continue to live and work, all while controlling disease spread, are crucial.

We first examined optimal reopening strategies with the testing ]colorred capacity fixed at *T*_*r*_ = 0.1. We made the observation that if *v*_*r*_ was high, a distinct set of 3 optimal strategies emerged, all of which allowed institutions to remain open for an optimal in-person experience of over 20 days. The first of the strategies involved a short opening, followed by a long closure, and then finally a long in-person session near the end of the semester (the semester length was fixed at *T* = 100 days). The second and third strategies were similar and corresponded to a shorter closure after the initial openings, followed by two moderate length in-person sessions (with closures). We suggest that the first strategy would be useful for universities in the sense that students often live far from home, and are unlikely able to return more than once after a closure (expensive in terms of time and money). However the second and third strategies might be useful for k-12 schools, in the sense that students typically live close to their schools and having weeks off followed by weeks on would allow a balance of in-person time that is important for parents, teachers, and students.

For low *T*_*r*_ and low *v*_*r*_, we noticed that distinct optimal peaks did not exist. Rather, the curves for *t*_*open*_ had different peaks for varying *v*_*r*_, and hence different reopening strategies. It should be noted that for *v*_*r*_ low enough (*v*_*r*_ < 0.3), the optimal in-person days always exceeds 70 days, and hence choosing any optimal strategy for these lower transmission rates will give you an excellent in-person experience (in terms of time spent in class).

We also explored increasing the testing capacity to a high value of *T*_*r*_ = 0.75. Here, we saw that the optimal number of in-person days increased to over 30 days, where two optimal strategies exist: one long closure followed by an extended open period, and an optimal strategy such that there were two shorter closures followed by two open periods. As in the first case when *T*_*r*_ is low, the first strategy might be better for universities, where the second could be more useful in a k-12 environment. We also note that for moderate values of *v*_*r*_ (*v*_*r*_ < 0.7) there are no closures such that the in-person experience is always 100 days.

A potential limitation of our modeling framework is that we did not explicitly take vaccination into account, as we assume all individuals in the system have the same vaccination status, a strategy taken by many US residential universities that require proof of vaccination upon arrival. However, in future work, the incorporation of differently susceptible individuals (e.g., vaccinated, unvaccinated, or boosted) into our model could be important, allowing an individual to go from susceptible to recovered without ever becoming sick or infecting others. In addition, this model extension could be used to address questions surrounding waning immunity, such that a vaccinated person can contract COVID again. This model addition could be particularly important when considering disease dynamics over longer time frames (longer than our 100 days, a typical semester length). To test whether we could ignore waning immunity in our model, we ran a simulation where we allowed a small proportion of recovered individuals to become susceptible again (at rate *γ*). Setting *γ* = 1/90 (from the range 1/90—1/30 days^−1^) had little effect on the overall dynamics in terms of the total number of infecteds at 100 days (results not shown). However, if the time frame is greater than 100 days, the infected numbers do increase, highlighting the potential importance of waning immunity over longer time periods.

In terms of robustness, our model suggests similar reopening strategies for a fixed testing capacity *T*_*r*_ *regardless* of the transmission rate *v*_*r*_ (when transmission is high). In fact, our model is robust to *all* model parameters, except *β*_1_ (the incubation rate) and *β*_2_ (the rate of recovery). The fact that our model is robust to *v*_*r*_ is very valuable, since throughout the pandemic, it has been quite difficult to accurately estimate disease transmission rate as it varies widely between variants and global location. This fact alone made it extremely difficult, especially at the start of the pandemic, to know when it was safe to reopen universities, businesses, etc. Our model provides a mechanism by which organizations can make more informed choices on reopening strategies, based on the knowledge of the incubation and recovery rates and their preferred testing strategy alone.

## Supporting information

S1 File Contains all the supporting tables and figures. (PDF)
