# Supplementary material for: Modeling optimal reopening strategies for COVID-19 and its variants by keeping infections low and fixing testing capacity
Source: PLoS One. 2022 Nov 9;17(11):e0274407. doi: 10.1371/journal.pone.0274407 (PMC9645599; doi:10.1371/journal.pone.0274407)
Supplement: S1 File — (PDF) [file pone.0274407.s001.pdf]

Supporting Information

Here we illustrate parameters for which our model given by equation (2) -(6) in the main text of the paper is robust and not robust. We show examples of model robustness using the same set of baseline parameters as outlined in Table 2 in the main text, and again here in Table S1. Figures S1 and S2 show that for a fixed testing capacity ($T_R = 0.1$), the model is not robust with respect to β_1 (the incubation rate) and β_2 (disease recovery rate from symptomatic state), respectively. In particular, notice the spread in the peaks for in-person day (t_{open}) for varying $\tilde{p}$ in Figures S1(a) and S2(a). Also, note that Figures S1(b) and S2(b) describe the corresponding histograms of all $\tilde{p}$ values that result in a number of days open within 5% of the maximum number of days open. Here, 1000 randomized values of β_1 and β_2 were used from their ranges summarized in Table 1 of the main text.

Baseline Parameters and Initial Conditions										
$S(0)$	$I_1^u(0)$	$I_1^a(0)$	$I_2(0)$	$R(0)$	f	β_1 (t^{-1})	β_2 (t^{-1})	β_3 (t^{-1})	$\tilde{\beta_1}$ (t^{-1})	$\tilde{\beta_3}$ (t^{-1})
0.99	0.01	0	0	0	0.85	0.143	0.06	0.05	0.143	0.05

Table S1. Base set of parameters and initial conditions for our model. We assume that 1% of the initial population is infected and are unaware of their infection.

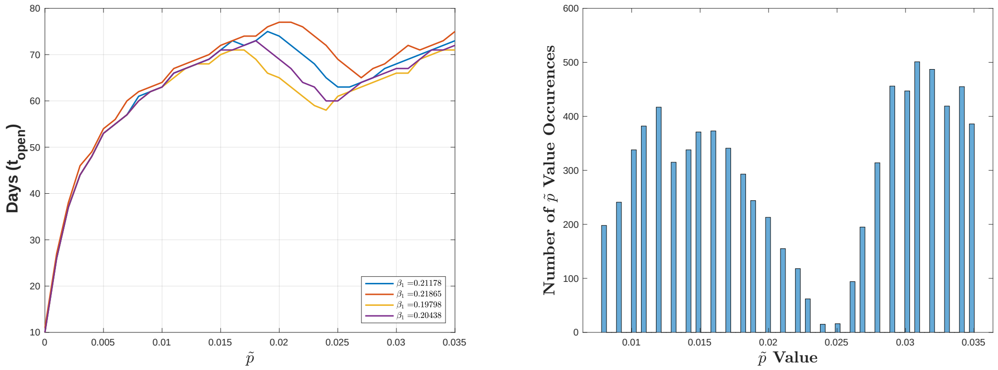

(a) In-person days t_{open} versus $\tilde{p}$ when varying β_1.

(b) Histogram of all $\tilde{p}$ values that result in a number of days open within 5% of the maximum number of days open. Here, 1000 randomized values of β_1 are chosen using a uniform distribution between 0.143 and 0.224.

Fig S1. Illustration that optimal in-person days t_{open} is not robust to changes in β_1. All model parameters used given in Table S1.

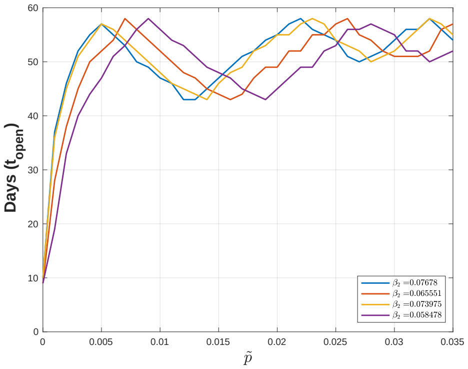

(a) In-person days t_{open} versus $\tilde{p}$ when varying β_2.

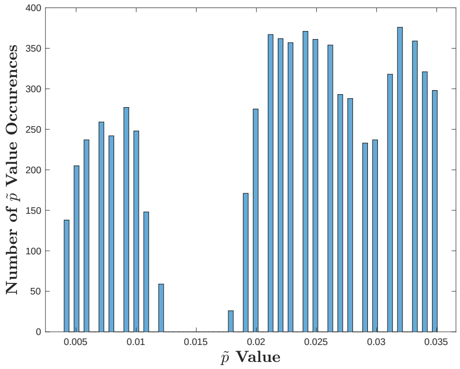

(b) Histogram of all $\tilde{p}$ values that result in a number of days open within 5% of the maximum number of days open. Here, 1000 randomized values of β_2 are chosen using a uniform distribution between 0.053 and 0.085.

Fig S2. Illustration that optimal in-person days t_{open} is not robust to changes in β_2. All model parameters used given in Table S1.

Recall in the main part of the text we already showed model robustness for high transmission rate v_r in Figures 4 and 8 for low ($T_r = 0.1$) and high ($T_r = 0.75$) testing capacities, respectively. Figures S3 to S5 show that for a fixed testing capacity ($T_R = 0.1$), the model it robust with respect to β_3, $\tilde{\beta}_3$, and $\tilde{\beta}_1$. Robustness holds regardless of the fixed testing capacity (results not shown). Notice well-defined peaks for in-person days (t_{open}) for varying $\tilde{p}$ in Figures S3, S4, and S5(a). In addition, and Figures S3, S4, and S5(b) describe the corresponding histograms of all $\tilde{p}$ values that result in a number of days open within 5% of the maximum number of days open. Here, 1000 randomized values of β_3, $\tilde{\beta}_3$, and $\tilde{\beta}_1$ were used from their ranges summarized in Table 1 of the main text.

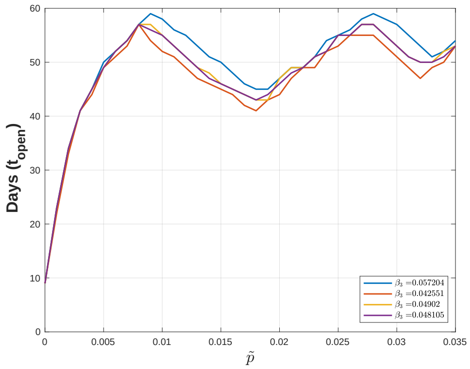

(a) in-person days t_{open} versus $\tilde{p}$ when varying β_3.

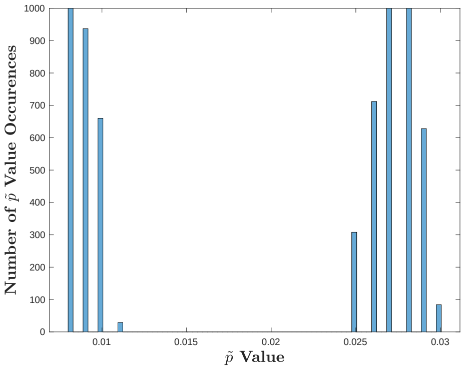

(b) Histogram of all $\tilde{p}$ values that result in a number of days open within 5% of the maximum number of days open. Here, 1000 randomized values of β_3 are chosen using a uniform distribution between 0.042 and 0.058.

Fig S3. Illustration that optimal in-person days t_{open} *is* robust to changes in β_3. All model parameters used given in Table S1.

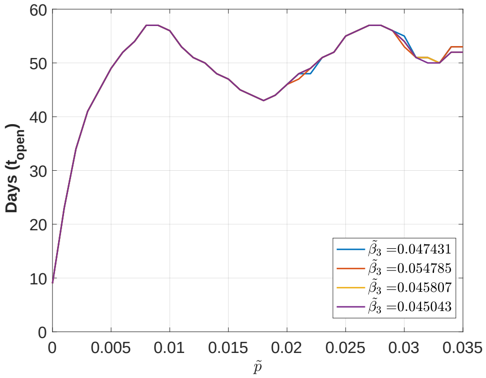

(a) in-person days t_{open} versus $\tilde{p}$ when varying $\tilde{\beta}_3$.

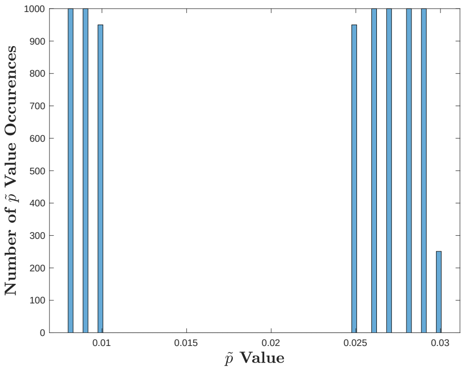

(b) Histogram of all $\tilde{p}$ values that result in a number of days open within 5% of the maximum number of days open. Here, 1000 randomized values of $\tilde{\beta}_3$ are chosen using a uniform distribution between 0.0.42 and 0.058.

Fig S4. Illustration that optimal in-person days t_{open} *is* robust to changes in $\tilde{\beta}_3$. All model parameters used given in Table S1.

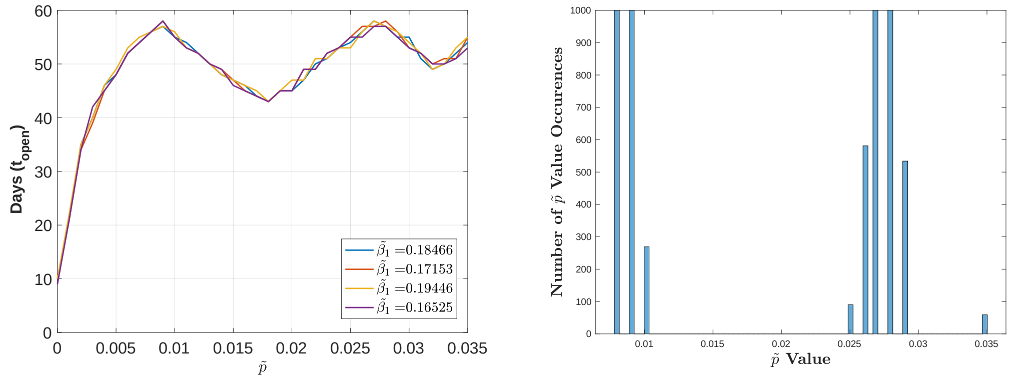

(a) in-person days t_{open} versus $\tilde{p}$ when varying $\tilde{\beta}_1$.

(b) Histogram of all $\tilde{p}$ values that result in a number of days open within 5% of the maximum number of days open. Here, 1000 randomized values of $\tilde{\beta}_1$ are chosen using a uniform distribution between 0.143 and 0.224.

Fig S5. Illustration that optimal in-person days t_{open} *is* robust to changes in $\tilde{\beta}_1$. All model parameters used given in Table S1.
